# Endovascular management of recurrent hemarthrosis of the knee: a case series

**DOI:** 10.1186/s42155-020-00135-0

**Published:** 2020-08-30

**Authors:** Richard Pow, Brett Fritsch, Richard Waugh, Chris Rogan

**Affiliations:** Royal Prince Alfred Hospital, 50 Missenden Road, Camperdown, Sydney, New South Wales 2050 Australia

**Keywords:** Embolization, Hemarthrosis, Geniculate artery, Knee

## Abstract

**Background:**

Recurrent hemarthrosis of the knee is an uncommon but potentially debilitating occurrence with multiple etiologies, including previous total knee replacement. The purpose of this study is to present data of a group of patients undergoing angiography and embolization for recurrent hemarthrosis of the knee. Patient characteristics, angiographic findings, safety and efficacy of the procedure are reported.

**Methods:**

A retrospective single centre review of patients undergoing angiography and embolization at a tertiary referral centre in Sydney, Australia from March 2006 to April 2018 was performed. A total of 25 patients undergoing a total of 29 procedures were identified (20 female, 5 male; mean age 67), the majority of which (23/25, 92%) had a history of total knee arthroplasty. Embolization was performed in 28 of the 29 procedures (97%). The embolic agent used was either polyvinyl alcohol particles (23/28), gelatin foam (3/28), detachable microcoils (1/28) or a combination of particles and coils (1/28).

**Results:**

The most commonly identified dominant vascular abnormality was periarticular synovial hypervascularity (23/25, 92%). A pseudoaneurysm was demonstrated in two patients (8%). Technical success (elimination of angiographic abnormalities) was achieved in 27 of 29 procedures (93%). There were 6 episodes of recurrence (25%) following a single embolization procedure, three of which were managed successfully with repeat embolization. There were no complications relating to skin or periarticular ischemia.

**Conclusion:**

Angiography and embolization is a safe and effective tool for the management of recurrent hemarthrosis of the knee following arthroplasty and should be considered first line treatment following failure of conservative management.

**Level of evidence:**

Level 4, Case Series.

## Background

Recurrent hemarthrosis of the knee is a potentially debilitating, but uncommon occurrence. In the setting of previous total knee arthroplasty (TKA), it occurs with a frequency of 0.3–1.6% (Bagla et al. 2013; Saksena et al. 2010), where it is associated with recurrent pain, joint-stiffness and poor post-operative recovery (Yoo et al. 2018). In this patient group, recurrent hemarthrosis is thought to most commonly relate to hypertrophy of vascular synovium and entrapment of hypertrophic synovium or fat between prosthetic components (Worland and Jessup 1996; Kindsfater and Scott 1995). Other causative or contributing factors in the post-operative setting may include implant malalignment, polyethylene wear and vascular abnormalities such as arterial pseudoaneurysm and arteriovenous fistula (Rukavina et al. 2010). In the absence of previous joint replacement, a variety of causes are recognized including anticoagulant therapy, a bleeding diathesis, pigmented villonodular synovitis (PVNS) and numerous systemic disorders.

Diagnosis is predominantly based on a combination of clinical symptoms and joint aspiration (Yoo et al. 2018). Conservative treatment is considered first line and typically includes joint aspiration, immobilization and cessation of anticoagulant or antiplatelet agents. For patients whom have failed conservative management, further treatment options include angiography with embolization or surgery, including synovectomy, either open or arthroscopic (Kindsfater and Scott 1995; Ohd et al. 2004).

Previous studies evaluating the utility of embolization for the management of recurrent hemarthrosis are mostly limited to case studies and small case series due to the rarity of this phenomenon (Kolber et al. 2016; Dhondt et al. 2009; van Baardewijk et al. 2019; Waldenberger et al. 2012; Guevara et al. 2018). It has however been recognized that angiography is capable of identifying and treating a vascular abnormality in almost all cases of recurrent hemarthrosis following TKA (Kolber et al. 2016; Dhondt et al. 2009). Rates of technical success with embolization are reported at 99–100% with clinical success rates of 80–93% (Kolber et al. 2016; Dhondt et al. 2009; van Baardewijk et al. 2019; Waldenberger et al. 2012; Guevara et al. 2018).

Given the paucity of data on the topic, this study seeks to further describe patient characteristics undergoing angiography and embolization for the management of recurrent hemarthrosis of the knee, as well as document procedure details, treatment safety and efficacy. It is hypothesised that our series will support the notion that embolization is a safe and efficacious intervention in this population group.

## Methods

### Study protocol

A retrospective review was performed with patients undergoing angiography and embolization for the management of hemarthrosis of the knee included in the study. Patients were identified through a keyword search of the radiology information and picture archiving and communicating systems (RIS/PACS). Medical records were reviewed to identify patient demographics, medical history and evidence of recurrence. Recurrence was based on clinical evidence of recurrent hemarthrosis by the time of chart review and assessment of re-intervention or repeat referral for further embolization.

Follow-up was with the referring surgeon and was not standardized. The mean time elapsed from the most recent angiographic procedure +/− embolization to the time of chart review was 64.1 months (range 8–154 months).

Ethics approval was obtained from the Sydney Local Health District Research and Governance Office.

### Patients’ characteristics

**Fig. 1:**
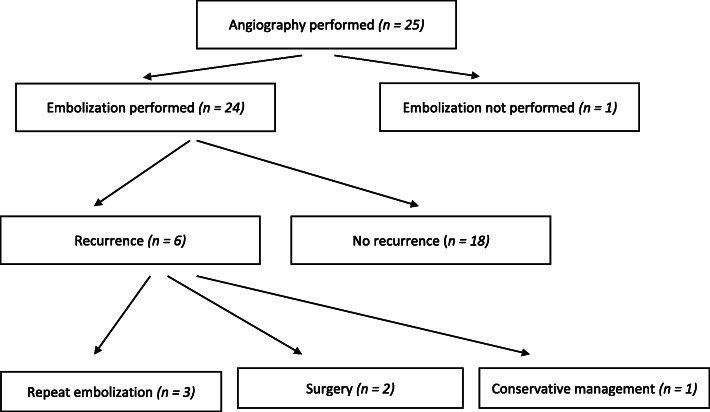
Study flowchart

All patients underwent angiography and embolization within the department of radiology at a single tertiary referral hospital in Sydney, Australia between the years 2006–2018. A total of 25 patients undergoing a total of 29 procedures met eligibility criteria and were included in the study (Fig. 1). Patients were excluded if there was a known or suspected infection of the prosthesis or a contraindication to angiography such as an uncontrollable bleeding diathesis or severe chronic kidney disease.

Five of the patients were male and 20 female. The mean age of patients at the time of initial procedure was 67 (Range: 22–84). Twenty-three patients had undergone previous TKA, 1 had previous anterior cruciate ligament reconstruction surgery and 1 had no surgical history. No clinically apparent cause for recurrent hemarthrosis was present in the two patients without a history of TKA. Sixteen patients were not taking any anti-platelet or anti-anticoagulant agent. Six patients were taking aspirin, 2 warfarin for previous pulmonary embolism (PE) or metallic valve replacement (1 each) and 1 rivaroxaban for previous PE.

Of the 23 patients with a history of TKA, the mean time interval from operation to initial embolization procedure was 17 months (Range: 2–54 months). Five patients had undergone arthroscopy +/− washout +/− synovial biopsy and debridement prior to the embolization procedure.

### Technique

Five procedures were performed under general anesthesia due to operator or patient preference. The remaining 24 procedures were performed under a combination of local anesthesia and conscious sedation. Fourteen of the procedures were performed via contralateral approach and 15 via an ipsilateral approach.

Approach, angiographic views, equipment selection, the choice of embolic agent and target vessels were at the discretion of the interventionalist and not standardized. In general, however, technique involved placement of a 5 Fr sheath in the common femoral artery followed by lower limb angiography including selective angiography of the femoral and popliteal arteries to identify pathologic peri-articular hypervascularity or a discrete vascular abnormality. Superselective catheterisation of the target vessel(s) was then performed with a microcatheter and the embolic agent delivered. The endpoint for embolization was reduction or elimination of peripheral hypervascularity ‘pruning’ and stasis in the target vessel, with care to avoid reflux.

## Results

### Angiographic findings and embolization

The dominant angiographic finding in 92% (23/25) of patients was that of increased vascularity about the knee joint with or without hypertrophy of the dominant feeding vessels. A pseudoaneurysm was visualised in 2 patients. This arose from a branch of the inferior lateral geniculate artery (ILGA) in both patients.

The majority (82%, *n* = 23/28) of embolization procedures used polyvinyl (PVA) particles (Contour; Boston Scientific) as the solitary embolic agent. Particle size ranged from 150 um to 1000 um, with almost all cases (96%, 23/24) using particles with a minimum size of 250 um. Gelatin foam (Gelfoam; Pfizer) was used in 3 patients (10%), delivered as a “slurry”.

**Fig. 2:**
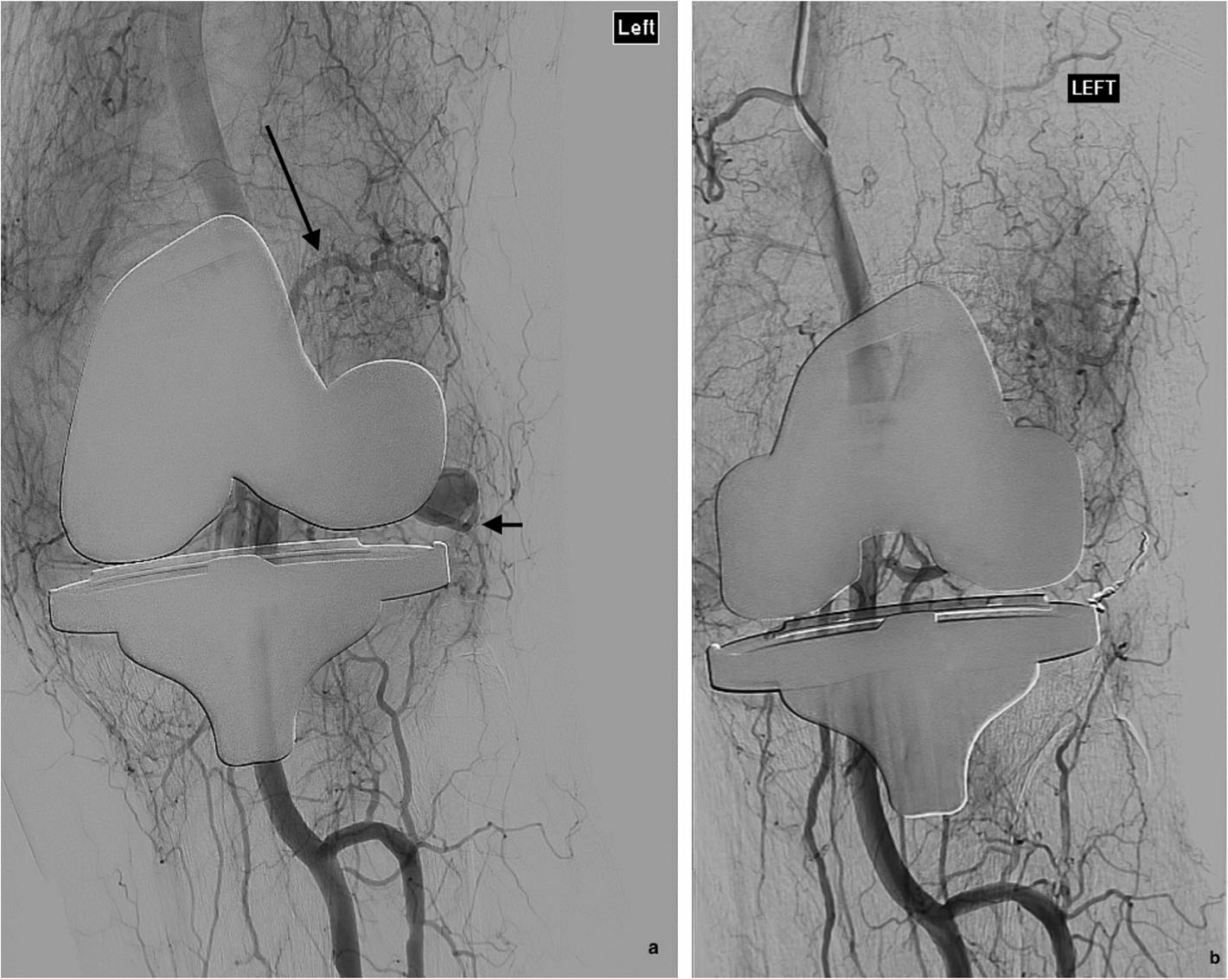
**a** Angiography demonstrates abnormally increased vascularity about the total knee replacement prothesis and a hypertrophic and tortuous superior lateral geniculate artery (long arrow). There is a pseudoaneurysm at the anterior aspect of the knee joint arising from the inferior lateral geniculate artery (short arrow). **b** There is successful occlusion of the pseudoaneurysm following coil embolization (2 x Ruby coils, 3 mm × 5 cm and 4 mm × 6 cm) of the feeding artery

In the two cases of pseudoaneurysm, coils (Ruby; Penumbra & VortX; Boston Scientific) were used as an embolic agent, one of which was combined with PVA particles for concomitant hypervascularity. In one case, microcoils were placed in the feeding vessel proximal to the origin of the aneurysm. In the remaining case, coils were placed in the feeding vessel across the neck of the pseudoaneurysm (Fig. 2). In both cases there was exclusion of the pseudoaneurysm following embolization.

Of the 28 procedures where embolization was performed, the mean number of vessels embolized was 2.25 (Range: 1–5) and the most commonly embolized vessels were the superior medial and lateral geniculate arteries, treated in 18 and 17 of 28 procedures respectively. See Table 1 for further detail.

**Table 1:** Characteristics and procedure details for patients undergoing angiography and embolization for recurrent hemarthrosis of the knee

Patient	Age	Gender	Post-TKA	Angiographic findings	Embolic agent	No of vessels embolised	Vessels embolised	Recurrence	Further management
1	82	M	Y	Pseudoaneurysm	Coils × 2	1	ILGA	No	–
2	78	F	Y	Hypervascularity	PVA particles	2	SLGA	Yes	Surgery (arthroscopic synovectomy and ablation)
							SMGA		
3	74	F	Y	Hypervascularity	PVA particles	1	SLGA	No	–
4	67	F	Y	Hypervascularity	PVA particles	1	SMGA	Yes	Repeat embolization
4^a^				Hypervascularity	PVA particles	5	SLGA	No	–
							IMGA		
							ILGA		
							MGA		
							Other		
5	22	M	N	Hypervascularity	PVA particles	3	SMGA	No	–
							ILGA		
							MGA		
6	61	F	Y	Hypervascularity	PVA particles	3	SMGA	No	–
							SLGA		
							MGA		
7	83	F	Y	Hypervascularity	PVA particles	3	SLGA	No	–
							IMGA		
							RB ATA		
8	84	M	N	Pseudoaneurysm & hypervascularity	Coils × 4	2	SMGA	No	–
					PVA particles		ILGA (pseudoaneurysm)		
9	71	F	Y	Hypervascularity	PVA particles	3	SMGA	Yes	Repeat embolization
							SLGA		
							MGA		
9^a^				Hypervascularity	PVA particles	5	SLGA	No	–
							SMGA		
							MGA		
							IMGA		
							RB ATA		
10	75	F	Y	Hypervascularity	Gelfoam	2	SMGA	No	–
							MGA		
11	76	F	Y	Hypervascularity	PVA particles	1	SMGA	No	–
12	71	F	Y	Hypervascularity	Gelfoam	2	SLGA	No	–
							MGA		
13	56	F	Y	Hypervascularity	PVA particles	3	SMGA	Yes	Repeat embolization
							SLGA		
							MGA		
13^a^				Hypervascularity	PVA particles	3	SMGA	No	–
							SLGA		
							ILGA		
14	55	F	Y	Hypervascularity	PVA particles	2	SLGA	No	–
							SMGA		
15	78	F	Y	Hypervascularity	PVA particles	2	SLGA	Yes	Cessation of anti-platelet agent
							SMGA		
16	81	F	Y	Hypervascularity	Gelfoam	1	ILGA	No	–
17	54	F	Y	Hypervascularity	PVA particles	1	SLGA	Yes	Surgery (revision TKA)
18	55	F	Y	Hypervascularity	PVA particles	3	SLGA	No	–
							SMGA		
							MGA		
19	77	F	Y	Hypervascularity	PVA particles	3	SLGA	No	–
							SMGA		
							MGA		
20	64	M	Y	Hypervascularity	PVA particles	2	SLGA	No	–
							MGA		
21	62	F	Y	Hypervascularity	PVA particles	1	SMGA	No	–
22	67	F	Y	Hypervascularity	PVA particles	1	MGA	No	–
23	62	F	Y	Hypervascularity	PVA particles	2	SLGA	No	–
							SMGA		
24	58	M	Y	Hypervascularity	–	–	–	–	–
25	60	F	Y	Hypervascularity	PVA particles	2	SGA	–	Repeat procedure due to vasospasm
							ASG		
25^a^				Hypervascularity	PVA particles	3	DGA / SMGA	No	
							ASG × 2		

*SLGA* superior lateral geniculate artery, *SMGA* superior medial geniculate artery, *MGA* middle geniculate artery, *ILGA* inferior lateral geniculate artery, *IMGA* inferior medial geniculate artery, *DGA* descending geniculate artery, *RB ATA* recurrent branch of anterior tibial artery, *ASG* accessory superior geniculate artery

^a^ Additional procedure on same patient

### Complications

There was no skin or peri-articular tissue ischemic complication and no complication relating to non-target embolization was observed. Three grade 1 complications occurred, with grading as per the CIRSE Classification System proposed by Filippiadis et al. 2017. There was a single case of non-flow limiting distal superficial femoral and popliteal artery dissection, treated with balloon angioplasty at the time of the procedure. There were two cases of small groin hematomas which required no further management. Vasospasm was encountered during one procedure which limited the ability to perform embolization to all target vessels and required a repeat procedure.

### Efficacy

Technical success, defined as devascularization of a focal vascular anomaly or intentional reduction of flow within a vascular bed, was achieved in 27 of the 29 procedures (93%). One of the procedures was limited by vasospasm, as described above. In another procedure, the interventionalist was unable to cannulate the target vessel to safely perform embolization (patient 24 in Table 1).

Recurrence of hemarthrosis occurred in 25% of patients following the initial embolization procedure (*n* = 6/24). Three patients underwent a repeat embolization due to recurrence with no further episodes of hemarthrosis encountered following repeat treatment. One patient encountered recurrence following commencement of an additional anti-platelet agent which was managed conservatively. Two patients with recurrence were managed operatively. One of these patients proceeded to arthroscopy where the cause of hemarthrosis was found to be an attritional tear of the posterior cruciate ligament (PCL). This was ablated and a synovectomy performed with no further episodes of recurrence. The other patient proceeded to revision TKA and had no further documented recurrence.

## Discussion

**Fig. 3:**
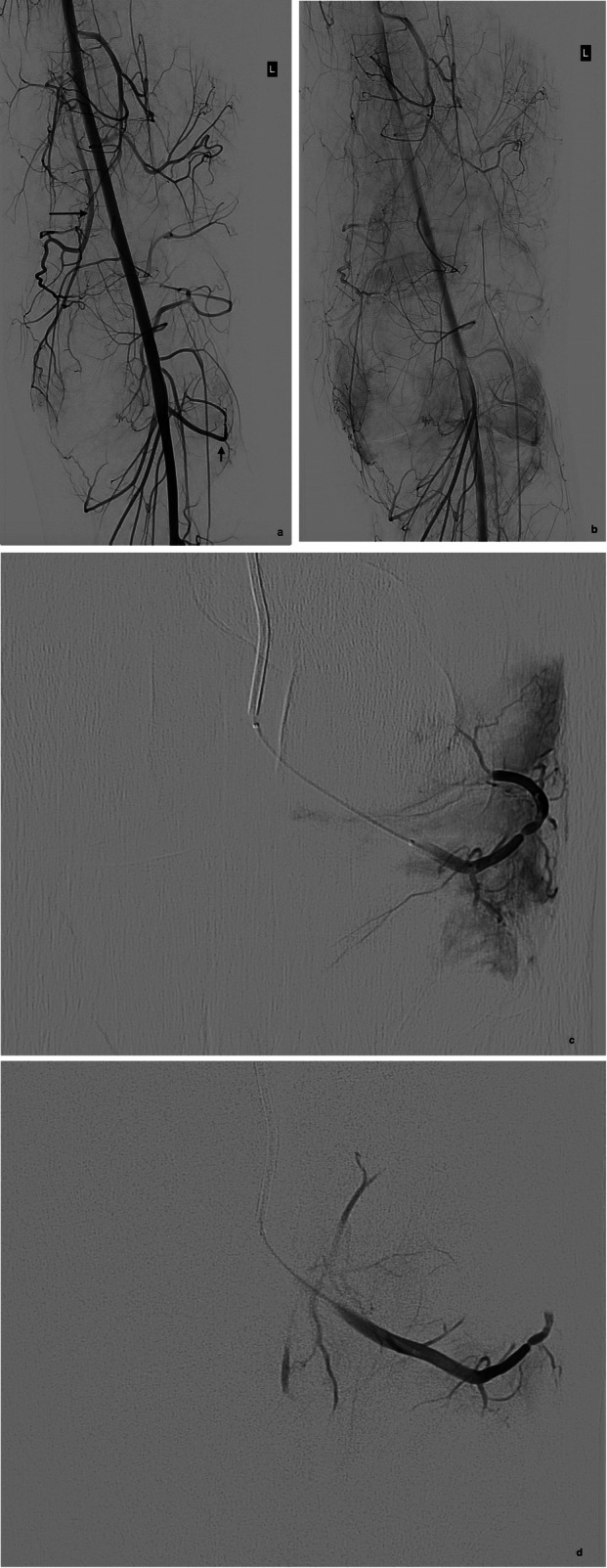
**a** Early and **b** late arterial phase angiography in a 22-year old patient with recurrent hemarthrosis and previous anterior cruciate ligament reconstruction demonstrates hypervascular synovium predominantly fed by the descending genicular / superior medial geniculate (long arrow) and inferior lateral geniculate arteries (short arrow). Selective angiographic runs of the inferior lateral geniculate artery **c** before and **d** after embolization demonstrates a marked reduction in vascularity following embolization with 150–250 μm PVA particles (pruning). Two further vessels were also treated (not shown)

Previous studies have documented that the most common angiographic abnormality in this patient group is that of peri-articular abnormal increased vascularity, with hypertrophy and/or tortuosity of feeding vessels, usually geniculate branches (Dhondt et al. 2009). This angiographic abnormality is thought to reflect the pathologically observed synovial hypertrophy and hypervascularity (Pham et al. 2013). As observed in other series (Kolber et al. 2016; Dhondt et al. 2009; van Baardewijk et al. 2019; Waldenberger et al. 2012), this was the most common angiographic abnormality (92%) encountered in our cohort (Fig. 3). This peri-articular hypervascularity is typically fed by several geniculate branches, accounting for the multiplicity of embolized vessels in our study. This synovial hypervascularity can also be seen in the setting of osteoarthritis of the knee, where early studies have shown promise in reducing vascularity and improving pain scores (Okuno et al. 2015; Bagla et al. 2020). Distinct vascular abnormalities such as pseudoaneurysms (Fig. 2) were a less common etiology in our cohort, observed in only 2 patients (8%), supported also by previous series (Kolber et al. 2016; Waldenberger et al. 2012).

As described above, we report a single case of an uncommon cause of hemarthrosis following TKA, that of an attritional tear of the PCL, which was confirmed and managed successfully operatively. This is an important orthopedic consideration in patients with a PCL-retaining implant and this accounted for a case of recurrence following embolization in our cohort (patient 2 in Table 1).

Cutaneous ischemia, usually transient, has been reported following geniculate artery embolization (Bagla et al. 2013; Guevara et al. 2018). There were no cutaneous or peri-articular ischemic complications in our cohort. This is despite 16% of patients (4/25) undergoing more than one embolization procedure. This is concordant with a series by van Baardewjik et al., who reported a cohort of 14 patients whom underwent a total of 24 embolization procedures, including 3 patients who each underwent 3 procedures. No complications relating to tissue ischemia were encountered and repeat embolization was associated with a high rate of clinical success (86%). Given the low rate of ischemic complications, we advocate embolization of all pathological geniculate arteries. The low ischemic complication rate also supports the notion that consideration can be given to repeat embolization in recurrent cases.

Furthermore, PVA particles represent the most commonly used embolic agent in our series (82%) as well as other major series (van Baardewijk et al. 2019; Waldenberger et al. 2012; Guevara et al. 2018). In our cohort, almost all (96%. *n* = 23/24) of the procedures involving the use of PVA particles used particles with a minimum particle diameter of 250 um. Particles smaller than this were generally avoided due to their potential to contribute to cutaneous ischemia. Increased rates of skin erythema or necrosis were reported in a systematic review by Kolber et al. with particles less than 300 μm, a phenomenon also reported in other anatomical sites (Koh et al. 2018; Khoury et al. 2017).

Three patients in our cohort underwent embolization with gelatin foam-based slurry (Gelfoam; Pfizer). This was at the discretion of the interventionalist and although successful embolization has been performed using gelatin based embolic agents previously (Yamagami et al. 2013), this is based on smaller scale trials and we prefer the use of PVA particles due to their more predictable and consistent particle size and non-temporary nature (Sheth et al. 2017). Calibrated microspheres would be expected to further improve predictable embolic size range due to more tightly calibrated particle sizing. Our sample size is too small to draw reliable conclusions regarding the optimal agent for embolization in this patient group.

Several mechanisms may be involved in recurrence following embolization. These include suboptimal initial embolization relating to a missed pathologic vessel or extensive synovial hypervascularity. Alternative explanations, which may account for cases of late recurrence, include collateral vessel formation or recanalization of previously embolized vessels (van Baardewijk et al. 2019).

We report a recurrence rate following a single embolization procedure of 25% (*n* = 6/24). Three of these patients were successfully managed with repeat embolization, reducing the rate of recurrence to 12.5% (*n* = 3/24). The 3 remaining patients were not considered for repeat embolization. Varying recurrence rates following a single embolization procedure have been reported in the literature, ranging from 6 to 50% (van Baardewijk et al. 2019; Waldenberger et al. 2012; Guevara et al. 2018), with recurrence rates declining to 3–14% following repeat embolization (van Baardewijk et al. 2019; Waldenberger et al. 2012). These figures are concordant with our series and variability in reported recurrence rates is likely accounted for small patient numbers and population heterogeneity.

The majority (23/25, 92%) of patients in our study had undergone previous TKA and thus this limits our ability to draw conclusions about the effectiveness of treatment in the absence of prior TKA. One of the largest series (Waldenberger et al. 2012) however reported similar and high rates of both technical (100%) and clinical (93%) success across a more heterogenous patient group, just over half of which had undergone prior joint replacement.

Our study has several limitations including its retrospective design and relatively small sample size. The reliance on medical records and documentation may have led to an underestimation of complication and recurrence rates as events may not have been captured if treatment was sought at an alternative facility or by an alternative clinician. A further limitation includes a lack of robust clinical follow-up including patient interviews and the lack of ability to quantify the overall improvement in function obtained by our treatment. It was unfeasible to obtain further follow-up due to the long-time interval between the time of embolization and data collection point.

## Conclusion

This study supports the notion that angiography is effective in demonstrating hypervascularity of the synovium and other vascular anomalies in cases of recurrent hemarthrosis of the knee. It appears that superselective arterial embolization is a safe and effective treatment option for recurrent hemarthrosis which should be an early management consideration following failure of conservative management.

## Data Availability

The authors conclusions are based on data and materials included in the manuscript. Permission to share this data is granted.

## Abbreviations

TKA: Total knee arthroplasty
PVNS: Pigmented villonodular synovitis
RIS/PACS: Radiology information systems and picture archiving and communication systems
PE: Pulmonary embolism
SLGA: Superior lateral geniculate artery
SMGA: Superior medial geniculate artery
ILGA: Inferior lateral geniculate artery
IMGA: Inferior medial geniculate artery
MGA: Middle geniculate artery
RB ATA: Recurrent branch of the anterior tibial artery
ASG: Accessory superior geniculate artery
DGA: Descending geniculate artery
PVA: Polyvinyl alcohol
PCL: Posterior cruciate ligament
